# Spontaneous formation of boron nitride nanotube fibers by boron impurity reduction in laser ablation of ammonia borane

**DOI:** 10.1186/s40580-022-00312-y

**Published:** 2022-05-12

**Authors:** Dong Su Bae, Chunghun Kim, Hunsu Lee, Omar Khater, Keun Su Kim, Homin Shin, Kun-Hong Lee, Myung Jong Kim

**Affiliations:** 1 Department of Chemical Engineering, Pohang University of Science and Technology, 77 Cheongam-ro, Nam-Gu, Pohang, Gyeongbuk 37673 Republic of Korea; 2 Department of Chemistry, Gachon University, 1342 Seongnam-daero, Sujeong-gu, Seongnam-si, Gyeonggi-do 13120 Republic of Korea; 3 Composite Materials Application Research Center, Korea Institute of Science and Technology, 92, Chudong-ro, Bongdong-eup, Wanju, Jeollabuk-do 55324 Republic of Korea; 4 Department of Mechanical Engineering, McGill University, 845 Rue Sherbrooke O, Montréal, QC H3A 0G4 Canada; 5 Security and Disruptive Technologies Research Centre, National Research Council Canada, 100 Sussex, Ottawa, ON K1A 0R6 Canada

**Keywords:** Boron nitride nanotubes, Laser ablation, Ammonia borane, Homogeneous nucleation, Spontaneous fiber formation, Pressure effect

## Abstract

Highly crystalline and few-walled boron nitride nanotubes (BNNTs) had been synthesized by laser ablation using only ammonia borane as a precursor. As a molecular precursor, ammonia borane supplied both B and N atoms with a ratio of 1:1, and BNNTs were formed via the homogeneous nucleation of BN radicals, not the growth from boron nano-droplets, which is a generally accepted growth mechanism of the laser-grown BNNTs. Owing to the absence of amorphous boron impurities, the van der Waals interaction among BNNTs became effective and thus a BNNT fibers was formed spontaneously during the BNNT synthesis. The BNNT growth and the subsequent fiber formation are found to occur only at high pressures of a surrounding gas. The mechanism behind the critical role of pressure was elucidated from the perspective of reaction kinetics and thermal fluid behaviors. A polarized Raman study confirmed that the BNNT fiber formed exhibits a good alignment of BNNTs, which implies great potential for continuous production of high-quality BNNT fibers for various applications.

**Supplementary Information:**

The online version contains supplementary material available at 10.1186/s40580-022-00312-y.

## Introduction

Boron nitride nanotubes (BNNTs) are a type of one-dimensional nanomaterial, and structurally identical with those of carbon nanotubes (CNTs); in BNNTs, carbon atoms are replaced by boron and nitrogen atoms composing hexagonal B-N lattices. Due to the structural similarity to CNTs, BNNTs also have excellent intrinsic properties, such as mechanical properties [1–3], high thermal conductivity [4, 5], thermo-mechanical stability [6] and oxidation resistance [7]. While CNTs consist only of covalent C–C bonds being either metallic or semiconducting [8] due to the overlap of π orbitals, BNNTs consist of polar covalent B-N bonds resulted from the differences in electronegativity of boron and nitrogen [9] and are electrically insulating with a band gap of ~ 5–6 eV. [10, 11] Thanks to the presence of boron element in BNNTs, BNNTs also have a high neutron absorption capability [12] and thus are highly regarded as excellent candidates for effective neutron shielding materials in aerospace applications.

BNNTs were theoretically predicted by Rubio et al*.* [10] in 1994 and first synthesized by Chopra et al*.* [13] in 1995 by arc-discharge. Since their first synthesis, there have been various attempts to synthesize BNNTs including laser ablation [14], ball milling [15] and chemical vapor deposition (CVD) [16]. For the BNNT growth, nitrogen or boron atom must be alternatively bonded after boron or nitrogen atom respectively, and thus the growth rate of BNNTs is expected to be slower than that of CNTs. Accordingly, the kinetic barrier in the BNNTs synthesis is higher than that of CNTs and thus their growth rate is also necessarily slow. One way to overcome such kinetic barrier is to supply high temperatures with high heat contents or to develop effective catalysts. Many research groups have attempted to optimize the CVD process through the development of effective catalysts for the BNNT synthesis, and a method, so-called boron oxide CVD (BOCVD), demonstrated one of the most promising results. In this process, boron powders react with metal oxides (e.g. MgO, FeO, Li_2_O) and generate B_2_O_2_ vapors, which subsequently react ammonia (NH_3_) to produce BNNTs [17, 18]. However, the precursor is limited to boron powders so the productivity and structural selectivity achieved in the CVD process for CNT production cannot be reached. A few years later, a couple of methods for synthesizing BNNTs with a highly crystalline has been developed by overcoming the kinetic barrier using the laser ablation [19] or thermal plasma [20] method.

Smith et al*.* [20] reported a high-temperature–pressure (HTP) method (vapor/condenser method) using a laser ablation technique where highly crystalline BNNTs continuously grow from nano-sized boron balls (i.e., seeds) through the reaction with ambient nitrogen gas. As seen in Haber–Bosch process [21], high energy barrier to dissociate the triple bond of nitrogen molecule was overcome by using catalyst, and Smith et al*.* used boron ball as a catalyst. Very recently, Kim et al*.* [22] elucidated the mechanistic details of the growth modes of BNNTs in the HTP process, and suggested a dual BNNT growth mode; the first mode is a root growth mode where a boron ball is continuously consumed through the reaction with ambient nitrogen gas to form BN radicals for the BNNT growth, while the second mode is an open-end growth in which heterogeneous nucleation of hexagonal boron nitride (h-BN) layers occurs on the outer surface of boron balls directly from BN radicals. In the both cases, the presence of boron balls is critical for the BNNT growth and a solid form of boron precursor (e.g., boron fiber or rod) has been often employed for the supply of nano-sized boron balls. In the previous method [19], therefore the formation of by-products such as amorphous boron is inevitable. The presence of such impurities significantly hinder the van der Waals interaction between BNNTs and thus suppress spontaneous formation of BNNT fibers which is a desirable form of BNNTs for many practical applications.

Herein, we report a laser synthesis of BNNTs using only ammonia borane, with reduced impurity contents, especially amorphous boron and the consequent *in-situ* formation of BNNT fibers. We propose using a molecular precursor, ammonia borane (H_3_N-BH_3_) that enables independent supply of B-N radicals one by one, analogous to carbon atoms in CNT synthesis. H_3_N-BH_3_ is a solid-state compound at room temperature composed of 6 hydrogen atoms bonded to a B-N bond. Since the bonding energies of B-H (330 kJ/mol) and N–H (314 kJ/mol) bonds are weaker than that of B-N (389 kJ/mol) bond, upon absorbing thermal energy H_3_N-BH_3_ gradually decomposes releasing hydrogen molecule and eventually turns into h-BN [23], which is an excellent precursor for the BNNT growth.

**Fig. 1:**
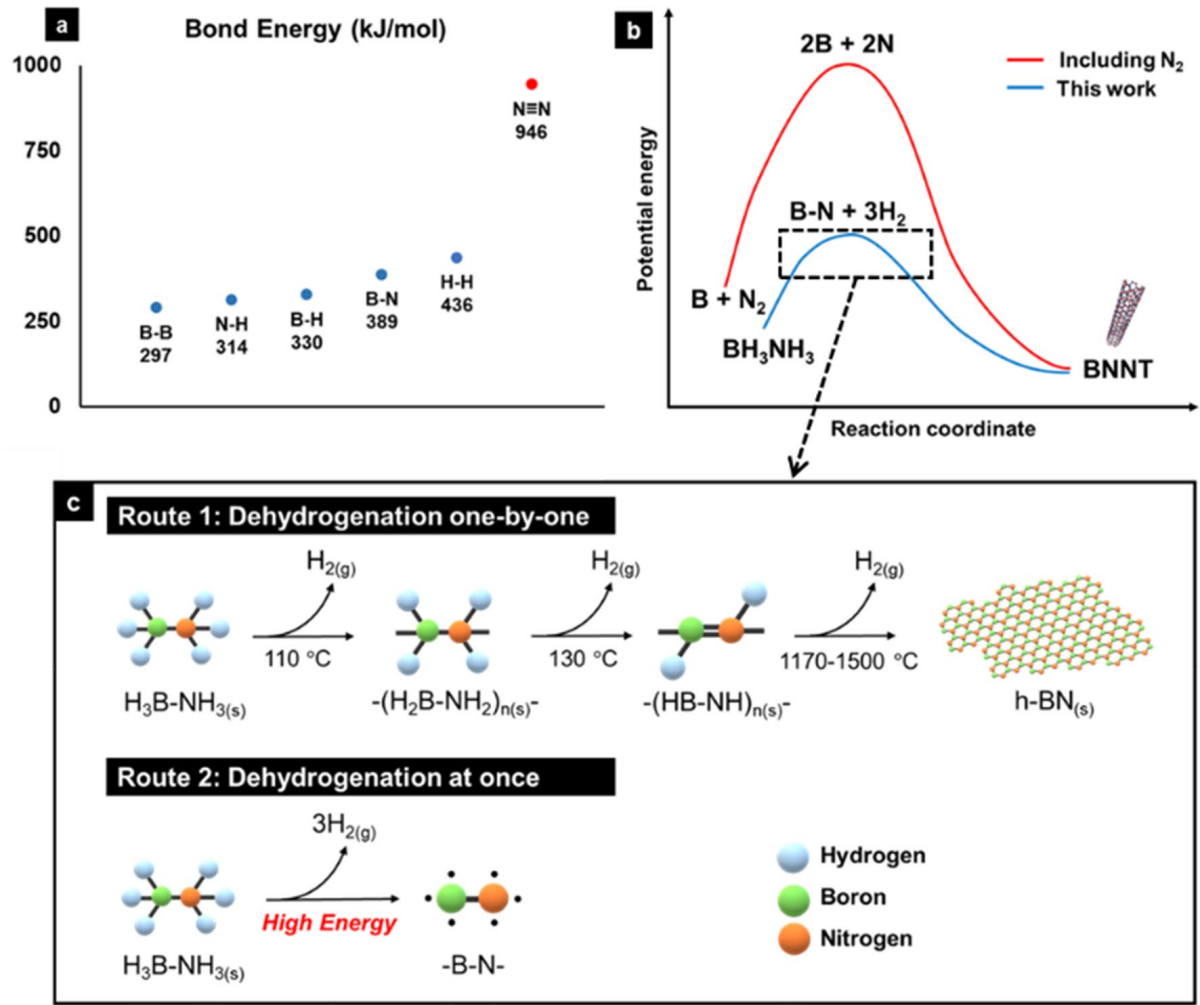
**a** Bond energy of bonds consists of B, N or H; **b** Conceptual energy diagram of BNNT synthesis starting from B + N2 and BH3NH3; **c** Two different ammonia borane pyrolysis routes according to the dehydrogenation process

There had been previous studies [24–26] that attempted to synthesize BNNT using the characteristics of ammonia borane described above. Templates or catalysts were used in the temperature range of 1450–1700 °C, and defective BNNTs with a radius of several hundred nm were synthesized. The BNNTs with a small radius and a small number of walls are required for industrial use have not been achieved. Those results were obtained because the domain of BN network (-B-N-) generated during the dehydrogenation of ammonia borane were fused to form a lot of defects before the BNNT formation. To overcome this, in this study, a laser was used to inject a much larger energy into ammonia borane instantaneously (Fig. 1c).

In the conventional approach of laser ablation synthesis of BNNTs, the ambient nitrogen gas must dissociate first to form B-N species through the reaction with boron balls, and thus the kinetic barrier of BNNT synthesis becomes lower (Fig. 1b) when H_3_N-BH_3_ is used as feedstock. In case the thermal decomposition of H_3_N-BH_3_ is performed rapidly using laser ablation, all the hydrogen atoms can be released at once leaving BN radicals behind, which can self-assemble into BNNTs in free space by clusterization.

In this work, we report that highly crystalline and few-walled BNNTs can be obtained with a minimum amount of amorphous boron impurities, which facilitates *in-situ* BNNT fiber formation via effective van der Waals interaction. The fact that amorphous boron impurity was rarely observed implies that the synthesis mechanism is different from the conventional understanding where nano-sized boron balls played a critical role in the formation of BNNTs. We have also studied the mechanism for the BNNT fiber formation with variations of surrounding gas pressure and gas species using both experimental and numerical approaches.

## Methods

### BNNT synthesis

**Fig. 2:**
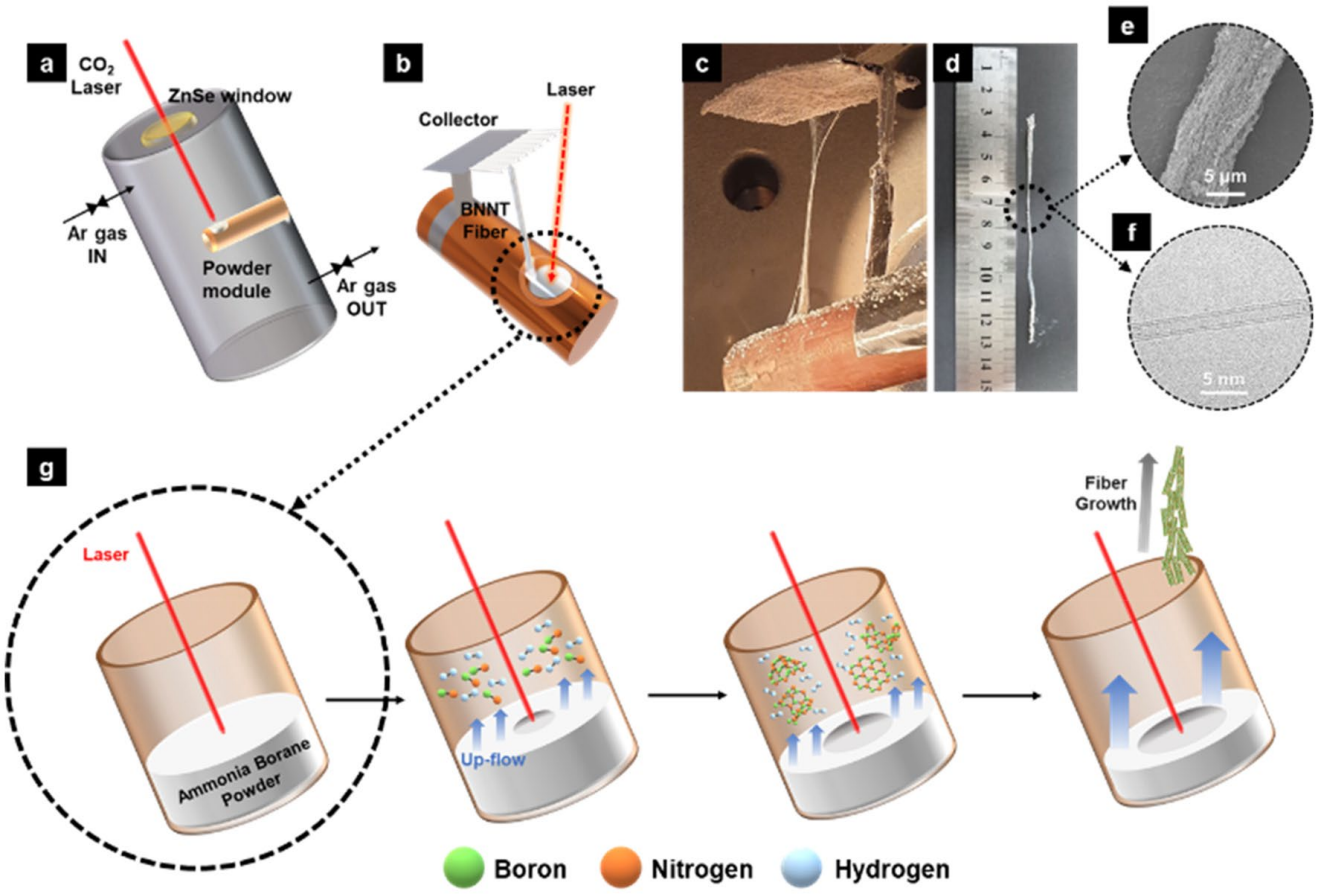
Schematic diagram of (**a**) BNNT synthesis equipment and **b** powder module; **c** Obtained BNNT fiber right after the reaction; **d** 10 cm long BNNT fiber; **e** SEM image of the BNNT fiber; **f** TEM image of the BNNT; **g** Schematic diagram of synthesis process

In order to proceed BNNT synthesis reaction by laser ablation, a continuous CO_2_ laser having an energy of 1000 W with a wavelength of 10.6 µm was employed. Figure 2A illustrates a schematic diagram of the experimental setup developed. BNNT synthesis was conducted in a cylindrical high-pressure chamber with a height of 140 cm and a diameter of 60 cm. A zinc selenide window attached to the upper part of the reaction chamber was used to selectively pass the CO_2_ laser while preventing external materials from entering. The gas inlet and outlet are located at the top and the bottom of the reaction chamber respectively, and the experiment was conducted under a constant chamber pressure with a fixed gas flow of nitrogen or argon. H_3_N-BH_3_ is a solid in a powder form at room temperature, and thus a powder container is required to use H_3_N-BH_3_ as a precursor. A powder module (Fig. 2b) made of copper is located at the center of the reaction chamber and cooled by water. A grid-type collector was placed above the powder module to collect BNNTs from the up-flow of reaction stream. A typical reaction proceeded for 30 minutes, and the temperature of the spot heated by the laser was maintained as 4000 K, which was measured from its black body radiation. Experiments were conducted with two different gases of argon (control experiments) and nitrogen under different chamber pressures of 2, 4, 8 and 12 bar. After a 30-min reaction, BNNT fibers of various lengths are obtained depending on the reaction condition. Figure 2c–d show a 10 cm-long BNNT fiber obtained. Currently, it is challenging to continuously supply the reaction precursor due to the limitations of the batch type experimental equipment; however, if the precursor were supplied continuously, a synthesis of BNNT fibers with longer lengths would be feasible.

### Numerical simulation

To understand the mechanistic pathway to formation of a BNNT fiber, computational fluid dynamics (CFD) simulations were performed. The laser ablation process is a very complicated process involving rapid materials transformation with a strong temperature gradient, and usually requires a three-dimensional (3-D) transient simulation. Due to the limited knowledge on the interaction between laser and an H_3_N-BH_3_ target, in this work, 3D steady simulations were performed with a constant ablation rate of the target. The diameter of the hot spot irradiated by the laser was assumed to be 1 mm and its temperate was fixed at 4000 K. To simulate a plume generated by ablation, a constant mass flow from the hot spot was considered. The mass flow rate was estimated from the average ablation rate observed in the experiment. The plume was assumed to be composed of only H_2_ and BN species with a ratio of 3:1. The thermodynamics and transport properties of the gases were obtained from the tabulated data calculated with a local thermodynamic equilibrium assumption while their pressure-dependent density was calculated using the ideal gas law. The temperatures of the power module and reactor wall were kept constant as 300 K. Lastly, the simulations were performed by solving conservation equations of mass, momentum, energy, along with the turbulence equations using the ANSYS/FLUENT software.

## Results and discussions

**Fig. 3:**
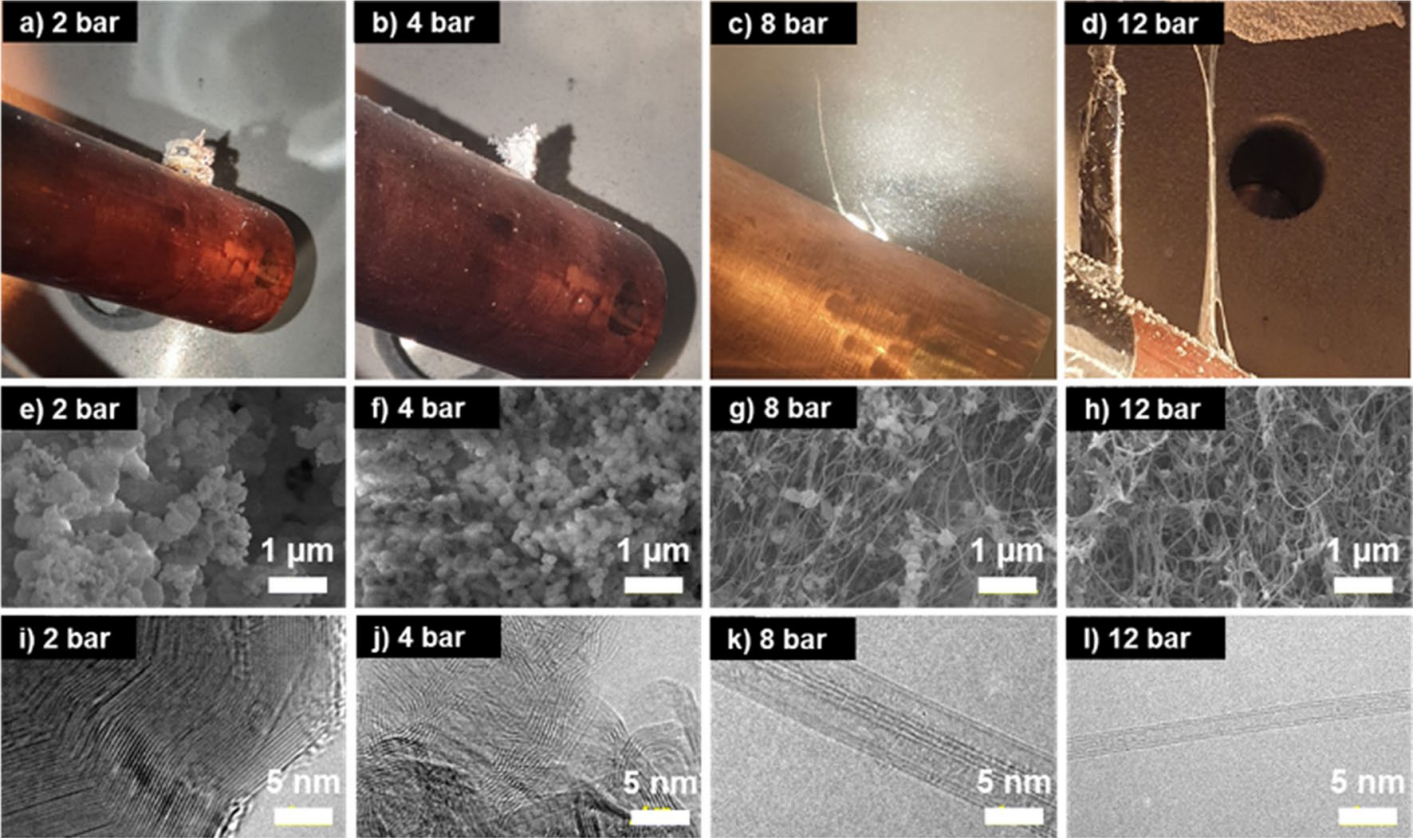
**a**–**d** Form, (**e**–**h**) morphology and **i**–**l** structure images of reaction products at 2, 4, 8 and 12 bar when the chamber is full of N2. BNNTs were dominant product at 8 and 12 bar and h-BN was dominant product at 2 and 4 bar

Different types of reaction products were obtained depending on the pressure inside the reaction chamber (Fig. 3a–d). At a reaction chamber pressure of 2 or 4 bar, white powdery material grew from the powder container to several centimeters height (Fig. 3a, b). Transmission electron microscopy (TEM (FEI technai at the Core-facility for Bionano Materials in Gachon University)) images show that the powder has a layer-by-layer structure (Fig. 3i, j) with a lattice distance of about 0.34 nm. Electron energy-loss spectroscopy (EELS) analysis revealed that B and N atoms were present across the sample with a ratio of 1:1, suggesting that the products are h-BN particles. However, at an elevated pressures of 8 or 12 bars, the main product was strands of white fibers stretching out from the powder container to the collector surface. TEM analysis shows that the fibers are composed of nano-scale fibrous materials that have a tubular structure (Fig. 3k, l). EELS analysis confirmed that the fibrous materials are composed of B and N atoms with a ratio of ~ 1:1 (Additional file 1: Fig. S1), suggesting that BNNTs are mainly produced at high pressures. Interestingly, unlike the products from the conventional laser ablation method, elemental B nanoparticles are rarely observed in this sample. In the previous studies [19] with a solid B target (e.g., B fibers), nano-sized B particles are often observed as by-products and proposed to serve as BNNT nucleation sites (i.e., BNNT seeds). However, in the current samples, majority of the impurity particles around BNNTs are h-BN particles (Additional file 1: Fig. S2) that exhibit the similar layer-by-layer structure (Fig. 3i, j) we observed with the white powder produced under the lower pressure of 2 or 4 bar. The size of h-BN impurities in the samples also depends on the pressure and decreases as the pressure increases (Fig. 3e–h). The absence of nano-sized B particles is intriguing as they are believed to play an important role in the BNNT nucleation and growth, and thus implies that a different growth mechanism is needed to explain the BNNT growth from ammonia borane.

**Fig. 4:**
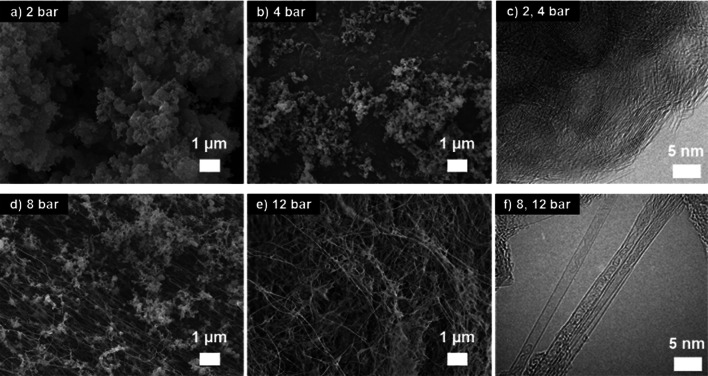
Morphology of reaction products by SEM analysis at (**a**) 2 bar, (**b**) 4 bar, (**d**) 8 bar and (**e**) 12 bar and structure of reaction products by TEM analysis at **c** 2 bar (similar at 4 bar) and (**f**) 8 bar (similar at 12 bar) when the chamber is full of argon. The same results were obtained when the chamber is full of nitrogen

In the previous studies of the synthesis of BNNTs by laser ablation, B and N atoms were independently supplied using solid boron and gaseous N_2_ molecule, respectively [19]. Although this study aimed to synthesize BNNTs by direct supply of BN units from H_3_N-BH_3_, we also speculated that additional N radical generated by dissociation of the surrounding N_2_ gas might have benefited the BNNT growth because the temperature of the hot spot is as high as 4000 K. A control experiment was performed with argon to investigate the effect of the surrounding gas, and almost same results were obtained; at pressures of 2 and 4 bar (Fig. 4a–c), white powdery materials were produced while BNNT fibers were formed at elevate pressures of 8 and 12 bar (Fig. 4d–f). It is also noted that the amount of reaction products were almost same compare with the N_2_ cases. This is probably because N_2_ molecule has a triple bond with high bonding energy (944.8 kJ/mol), and thus the amount of nitrogen gases decomposed into nitrogen atoms was extremely small even at a high temperature of 4000 K. The control experiment demonstrated that the type of the surrounding gas is not critical for the BNNT synthesis, and ammonia borane itself is sufficient to supply B and N atoms at a 1:1 ratio for the BNNT growth.

Our main finding from the parametric study can be summarized as follows: (i) the morphology of the main reaction product changes from h-BN to BNNTs as the chamber pressure increases; (ii) nano-sized B droplets are not produced if ammonia borane is used as feedstock; (iii) the type of the surrounding gas is not critical for the BNNT growth as it seems not participating in the BNNT synthesis reaction. Obviously, these findings are not consistent with the growth mechanism proposed by Kim et al. (i.e., dual growth mode) for the laser-grown BNNTs, and thus implies different pathways in the BNNT nucleation and growth by ammonia borane.

In ammonia borane, N-B bond is stronger than B-H and N–H bonds. Upon thermal energy absorption, the relatively weak B-H and N–H bonds are expected to break first and release H atoms. When the temperature increases gradually from room temperature to 1500 °C, ammonia borane forms polyaminoborane (-(BH_2_-NH_2_)-_n_) and polyiminoborane (-(BH-NH)-_n_) in consecutive order through dehydrogenation process. Such intermediate species eventually turn into h-BN through further hydrogen release. However, a rapid temperature increment by laser irradiation may result in an immediate decomposition of ammonia borane into various gaseous species including BN, BH, NH, and BNH radicals, rather than bulk h-BN. In this case, BN radicals are expected to be the most dominant species among the various chemical species due to its strongest bonding energy. We also suggest that the BN radicals formed do not subsequently decompose into elemental B and N atoms because elemental boron particles were seldom observed from our samples. In the previous study [19], TEM images of BNNT synthesized often show BNNTs with B nano-droplets at their tips. However, in this study, such nano-droplets were never seen from TEM analysis (Additional file 1: Fig. S3). This argument is also supported by thermogravimetric analysis (TGA) of our samples where the mass gain by B_2_O_3_ formation is not significant during oxidation (Additional file 1: Fig. S4). The mass increment from 650 °C to 800 °C in the TGA graph is due to the oxidation of elemental boron particles while the mass gain around 900 °C might be associated with the oxidation of defective BN by-products, such as amorphous or turbostratic BNs because crystalline h-BN materials start to oxidize from 950 °C. The elemental boron content in this sample is estimated as low as 1.35 wt. %. The absence of elemental boron partially explains why the BNNT growth from ammonia borane is less sensitive to the type of the surrounding gas. Unlike the conventional process, the re-nitridation of elemental boron by N radicals or excited N_2_ molecules (e.g., vibrationally excited N_2_ (ν) or N_2_^+^ ions) are not an essential step in this case. Our study found that BNNTs seem to grow directly from BN radicals when ammonia borane is used as feedstock, but at the same time it raises an important question of how BNNTs nucleate and grow directly from BN radicals in free space.

**Fig. 5:**
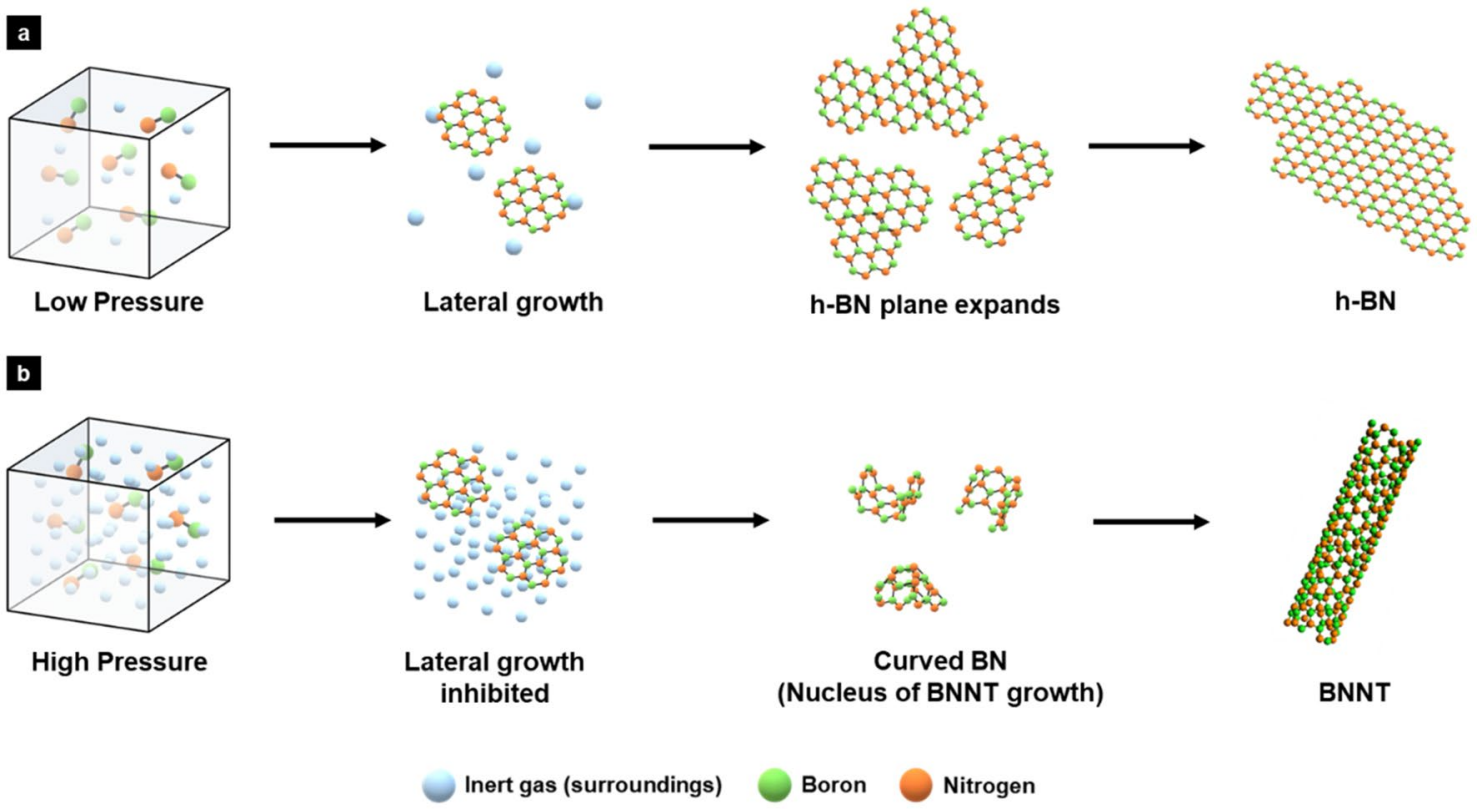
Mechanism of nucleus formation of BNNT synthesis. **a** At low pressure, B-N radicals bond each other quickly, and h-BN forms in a lateral direction. **b** At high pressure, lateral growth of h-BN is inhibited by surrounding gas, so curved h-BN forms, and can nucleate BNNT synthesis

BN radicals formed can be a major precursor for continuous of growth of both h-BN and BNNTs. The fact that the morphology of the main product is determined by the reaction pressure implies that the BNNTs growth is not dictated by chemistry but by kinetics. Since h-BN has a planar structure, BN radicals could form h-BN via lateral growth as illustrated in Fig. 5a. When the pressure of the surrounding gas increases, the concentration of BN radical decreases and consequently the lateral growth facilitated via collisions among BN radicals may be inhibited by more frequent collisions with surrounding gas molecules (Fig. 5b). The rate of lateral growth of h-BN is inversely proportional to the pressure of the surrounding gas. As the growth speed slows down, the size of the h-BN fragment decreases which is in line with our experimental observation, and also the number of dangling bonds per area increases, making them more unstable. The latter can facilitate folding or zipping of h-BN fragments in order to reduce the number of unstable dangling bonds (Fig. 5b).

At high pressures, h-BN fragments also gain enough energy by collision with surrounding gases and can overcome the strain-energy barrier required for curvature formation (Fig. 5b). This phenomenon has also been reported in the kinetics of fullerene formation [27]. Laser was applied to a rotating graphite disk and carbon species were vaporized into a stream of helium (He), cooled and partially equilibrated during the expansion. When the pressure of He stream was less than 1 atm, the number of C atoms per cluster was distributed in the range of 50 to over 90; however when the pressure increased to 10 atm, clusters of 60 carbon atoms (i.e., fullerene) were selectively obtained. When the hot ring clusters are remained in contact with high-density He, they equilibrate towards the most stable species through two- and three body collisions, which appears to be a unique cluster containing 60 atoms.27 This phenomenon can be adapted to explain the pressure effect of this study and BNNT growth from BN radicals at high pressures. At high pressures, C clusters are subjected to frequent collisions with the surrounding gases which provides sufficient energy to overcome the kinetic barrier that originates from the curvature strain. Therefore, they tend to form thermodynamically-favorable species of a spherical fullerene. However, in the case of BN, frustration of bonding occurs when a B-B bond or an N–N bond is created in the pentagonal structure [28]. The energetically-favorable curved structure is a tubular structure (i.e., nanotube) rather than a BN fullerene. Starting from a curved seed as illustrate in Fig. 5b, B and N atoms form a nanotube at high pressures by eliminating energetically-costly dangling bonds in the edges. In this work, the curved B-N structure (Fig. 5b) is proposed as the nucleus of BNNT formation (i.e., homogeneous nucleation). For the growth of BNNT, BN radicals are continuously added to the curved h-BN seed and a BNNTs grow in the axial direction. BN radicals can also participate in growing other h-BN species when the edges of the curved seed are not commensurate with formation of a tubular structure. Such by-products become main impurities of the product (Additional file 1: Fig. S2).

**Fig. 6:**
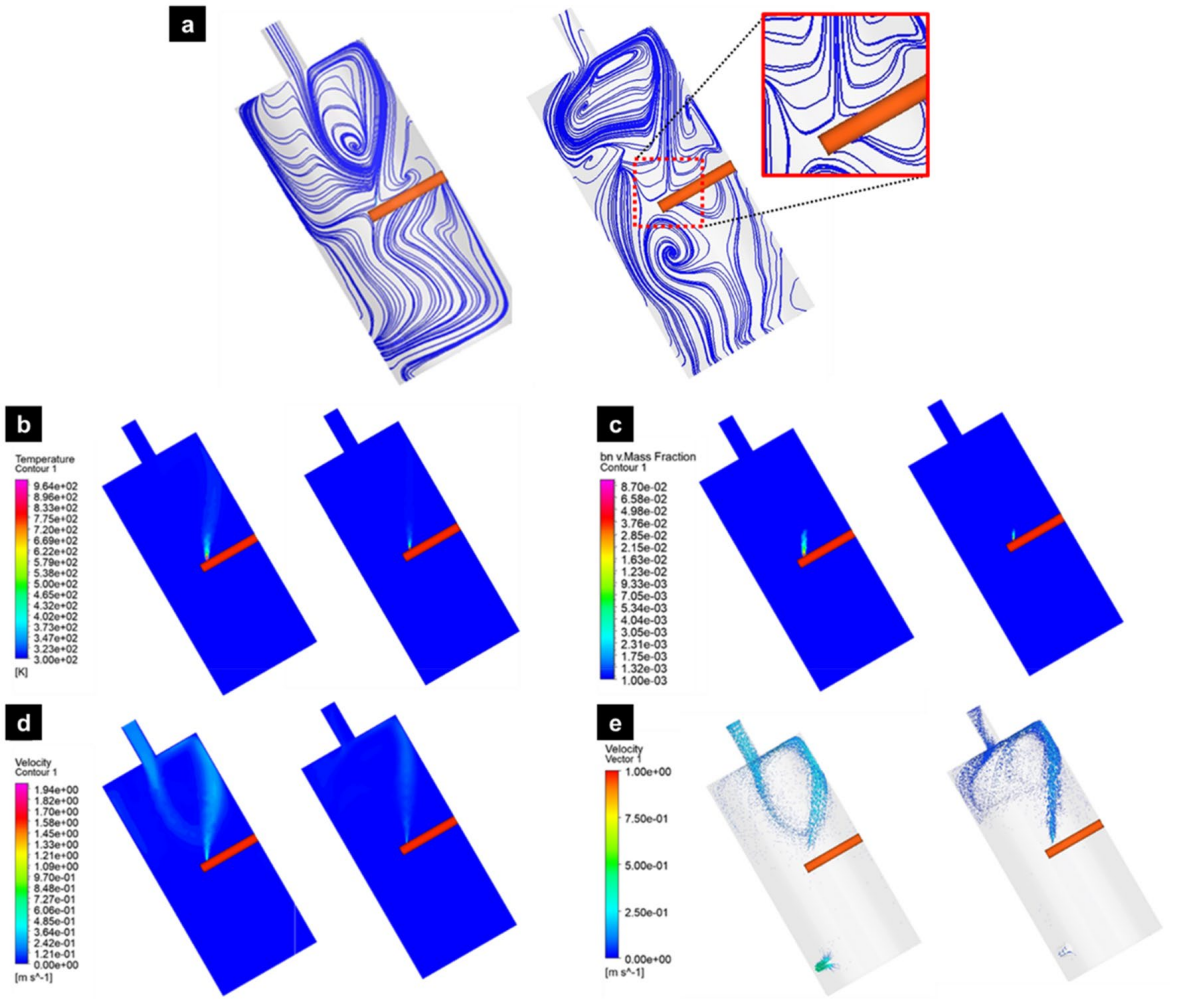
Thermo-fluid simulation results in the reaction chamber at (left) 2 and (right) 12 bar. **a** Streamlines, **b** temperature distribution, **c** BN mass fraction, **d** velocity distribution, **e** velocity vector

Results of the thermo-fluid simulation such as temperature, BN mass fraction, and velocity fields along with streamline analysis, are present in Fig. 6. As the surrounding gas pressure increases, it was predicted that the high temperature zone shrinks and the BN concentration also decreases in the reaction zone (Fig. 6b). This is a pure pressure effect caused by adding more surrounding gas. The velocity distributions (Fig. 6d) predict an up-flow formation due to the evaporation of ammonia borane and the temperature difference between the laser spot and the surroundings (i.e., buoyancy force). The flow field pattern also changes slightly with the pressure. In the case of low pressure (e.g., 2 bar), the gas velocity flowing in from the upper part is larger than that of the high pressure (e.g., 12 bar) case, thus the BN precursors or BN debris generated may re-enter the reaction zone (Additional file 1: Fig. S5) which increases the amount of BN in the reaction zone. On the other hand, in the case of 12 bar, the velocity of the gas from the upper part is reduced and thus the possibility of re-entering into the reaction zone seems relatively small (Additional file 1: Fig. S5). The simulation results support our discussion that h-BN growth may slow down at high pressures due to the reduction of BN radicals in the reaction zone. It was also observed that the direction of the up-flow is fairly consistent with the direction of BNNT fiber formation. It seems that the up-flow from the hot spot helps in-situ fiber formation by confining and directing BNNTs toward the flow direction (i.e., flow-driven alignment).

**Fig. 7:**
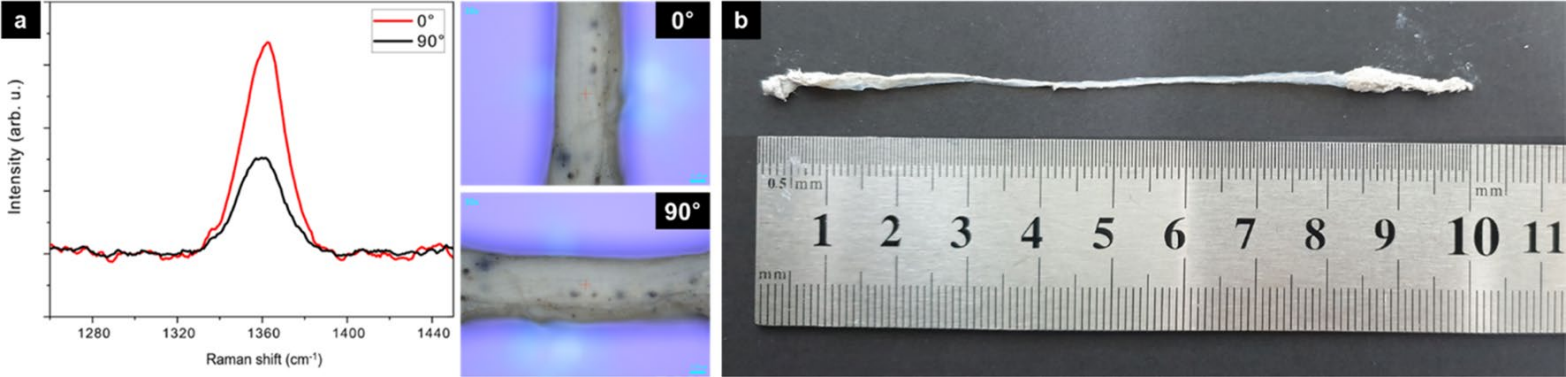
**a** Raman spectra for the BNNT fiber at 0° and 90° under the VV mode. **b** As-grown 10 cm long BNNT fiber

The degree of alignment of BNNT fibers was also analyzed by the Raman signal intensity (I) in VV mode (Fig. 7a). BNNT fibers show a peak near 1360 cm^−1^, attributed to the E_2g_ vibrational mode in the h-BN sheet [29]. The Raman signal intensity was maximum at 0° while minimum at 90°. Previous research has shown that the intensity I in VV mode is [30]

1 $$I_{\theta } = \, A \, cos^{4} \theta \, + \, B,$$

_0°_/I_90°_ = 2.1. These results show a degree of alignment like the I_0°_/I_90°_ = 2.2 reported previously for BNNT alignment [31] The data used for calculation are average values obtained by measuring each sample at least three times. From the data, we can conclude that the well-aligned BNNT fiber has been formed from the method we report.

## Conclusion

We report the synthesis of BNNTs by laser ablation using a molecular precursor, ammonia borane (H_3_N-BH_3_) that supplies BN radicals one by one as BNNT precursors. Unlike the conventional method, highly-crystalline BNNTs were obtained with a minimal amount of amorphous B impurities. This result implies that the BNNT growth mechanism in this work is different from the conventional high temperature method where nano-sized B droplets play an important role in the BNNT nucleation. By considering kinetics and thermal fluid dynamics, we found that at high pressures (> 8 bar) of argon gas, BNNTs could nucleate and grow directly from BN radicals released from ammonia borane, in the absence of boron droplets or pre-existing catalyst nanoparticles (i.e., homogenous nucleation). This greatly reduces B impurities in the reaction stream, and thus enables *in-situ* formation of BNNT fibers via van der Waals interactions among BNNTs. The BNNT fibers reported here has high potential for various applications similar to CNT fibers after desired reinforcement to increase the strength of the fiber. We leave it as a future work.

## Supplementary Information

**Additional file 1: Figure S1.**(a) EELS and (b) EDS analysis of the BNNTs.** Figure S2.** Structure of impurities analyzed by HR-TEM. Most of the impurities are made of h-BN layers. Figure S3. Structure of tube ends analyzed by HR-TEM. Most of the tube ends are closed without boron droplets.** Figure S4.** Thermogravimetric analysis of as-grown BNNTs. Amorphous boron content is 1.35 wt.%.** Figure S5.** Streamlines calculated in the reaction chamber at (left) 2 and (right) 12 bar. Red arrows indicate a potential pathway of BN precursors or debris formed.

## Data Availability

The datasets used and/or analyzed during the current study are available from the corresponding author on reasonable request.

## Abbreviations

BNNT: Boron Nitride Nanotube
CNT: Carbon Nanotube
h-BN: Hexagonal Boron Nitride
