# Supplementary material for: Spontaneous formation of boron nitride nanotube fibers by boron impurity reduction in laser ablation of ammonia borane
Source: Nano Converg. 2022 May 12;9:20. doi: 10.1186/s40580-022-00312-y (PMC9098712; doi:10.1186/s40580-022-00312-y)
Supplement: Supplementary file 1 — Additional file 1: Figure S1.(a) EELS and (b) EDS analysis of the BNNTs. Figure S2. Structure of impurities analyzed by HR-TEM. Most of the impurities are made of h-BN layers. Figure S3. Structure of tube ends analyzed by HR-TEM. Most of the tube ends are closed without boron droplets. Figure S4. Thermogravimetric analysis of as-grown BNNTs. Amorphous boron content is 1.35 wt.%. Figure S5. Streamlines calculated in the reaction chamber at (left) 2 and (right) 12 bar. Red arrows indicate a potential pathway of BN precursors or debris formed. [file 40580_2022_312_MOESM1_ESM.docx]

**Spontaneous Formation of Boron Nitride Nanotube Fibers by Boron Impurity Reduction in Laser Ablation of Ammonia Borane**

Dong Su Bae,**^a^** Chunghun Kim,**^b^** Hunsu Lee,^c^ Omar Khater,^d^ Keun Su Kim,^e^ Homin Shin,^e^ Kun-Hong Lee**^a^*** and Myung Jong Kim ^b^*

^a^Department of Chemical Engineering, Pohang University of Science and Technology, 77 Cheongam-ro, Nam-Gu, Pohang, Gyeongbukn 37673, Republic of Korea

^b^Department of Chemistry, Gachon University, 1342 Seongnam-daero, Sujeong-gu, Seongnam-si, Gyeonggi-do, 13120, Republic of Korea

^c^Composite Materials Application Research Center, Korea Institute of Science and Technology, 92, Chudong-ro, Bongdong-eup, Wanju, Jeollabuk-do 55324, Republic of Korea

^d^Department of Mechanical Engineering, McGill University, 845 Rue Sherbrooke O, Montréal, QC H3A 0G4

^e^Security and Disruptive Technologies Research Centre, National Research Council Canada, 100 Sussex, Ottawa, ON K1A 0R6, Canada

E-mail address for corresponding authors

Myong Jong Kim: [myungjongkim@gachon.ac.kr](mailto:myungjongkim@gachon.ac.kr)

Kun-Hong Lee: [ce20047@postech.ac.kr](mailto:ce20047@postech.ac.kr)


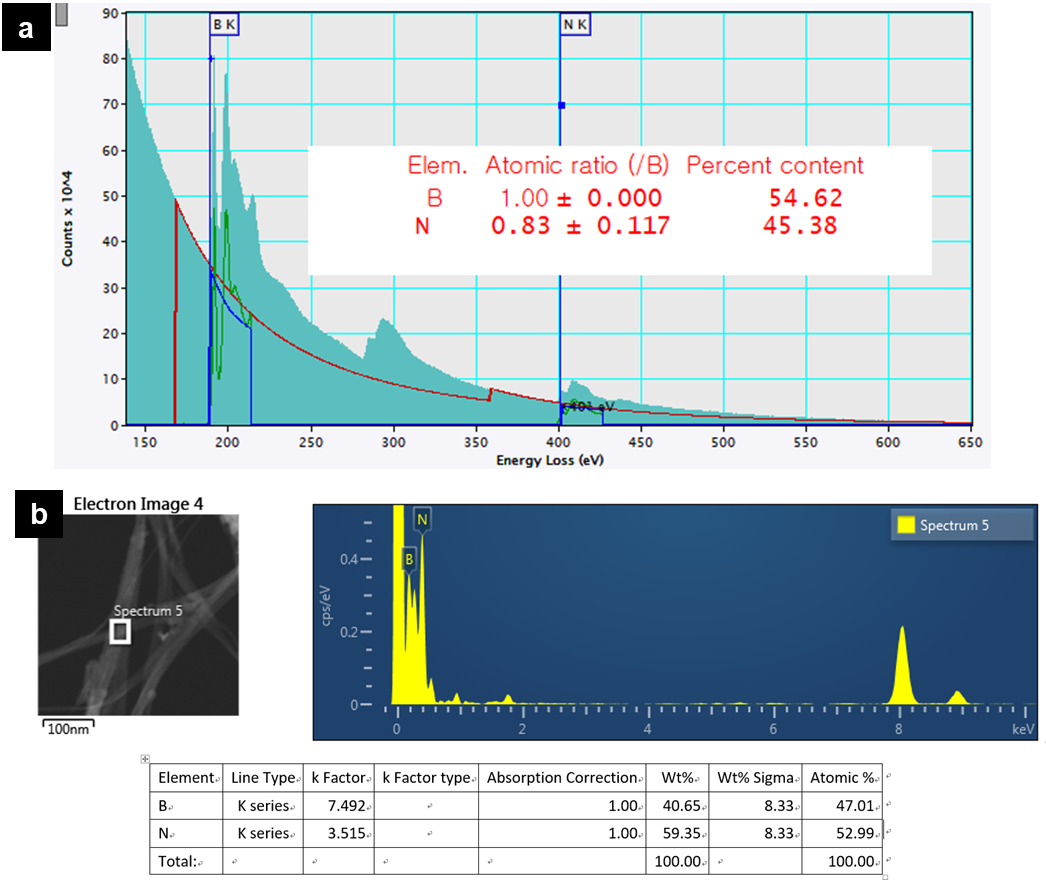


Figure S1. (a) EELS and (b) EDS analysis of the BNNTs


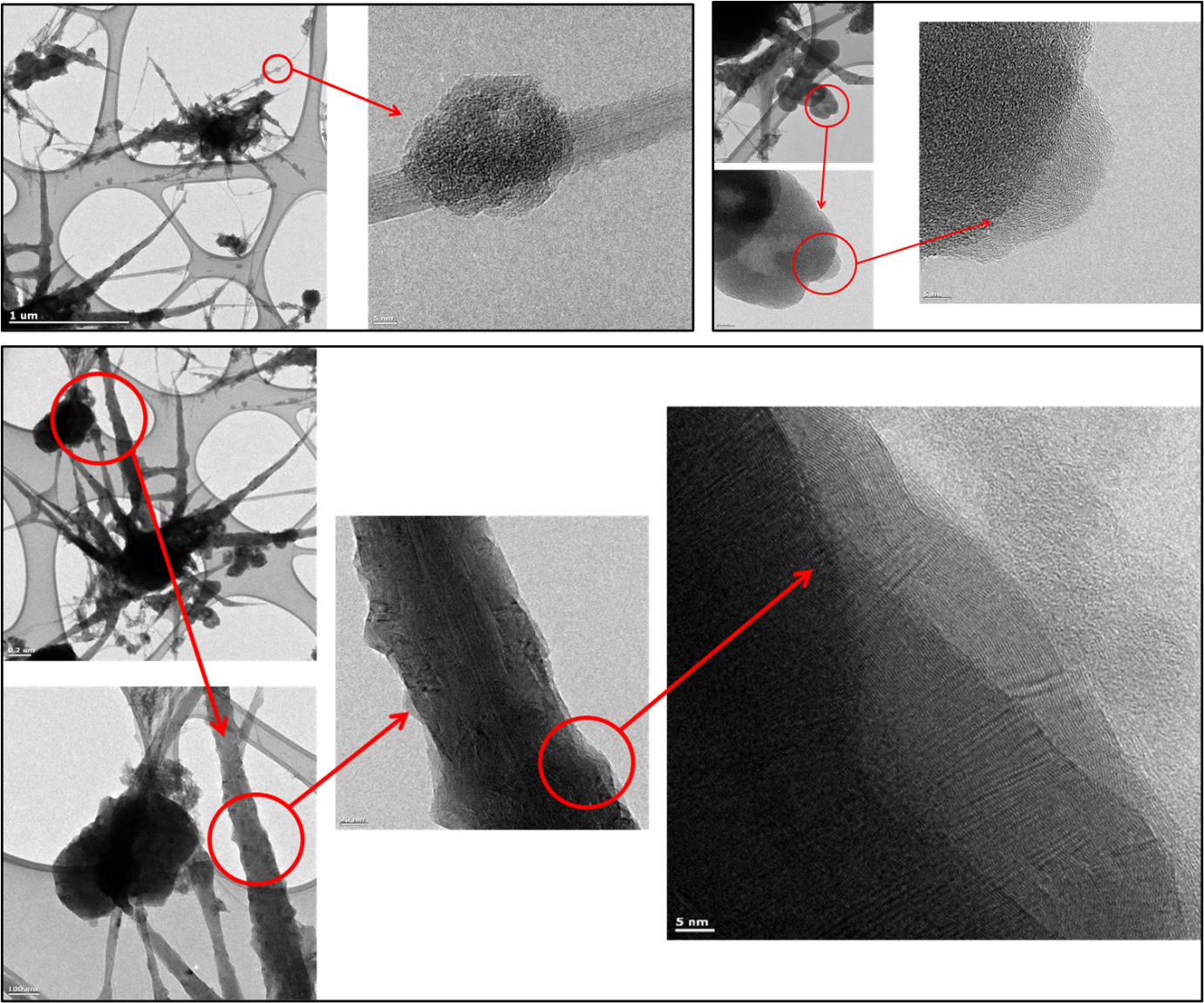


Figure S2. Structure of impurities analyzed by HR-TEM. Most of the impurities are made of h-BN layers.


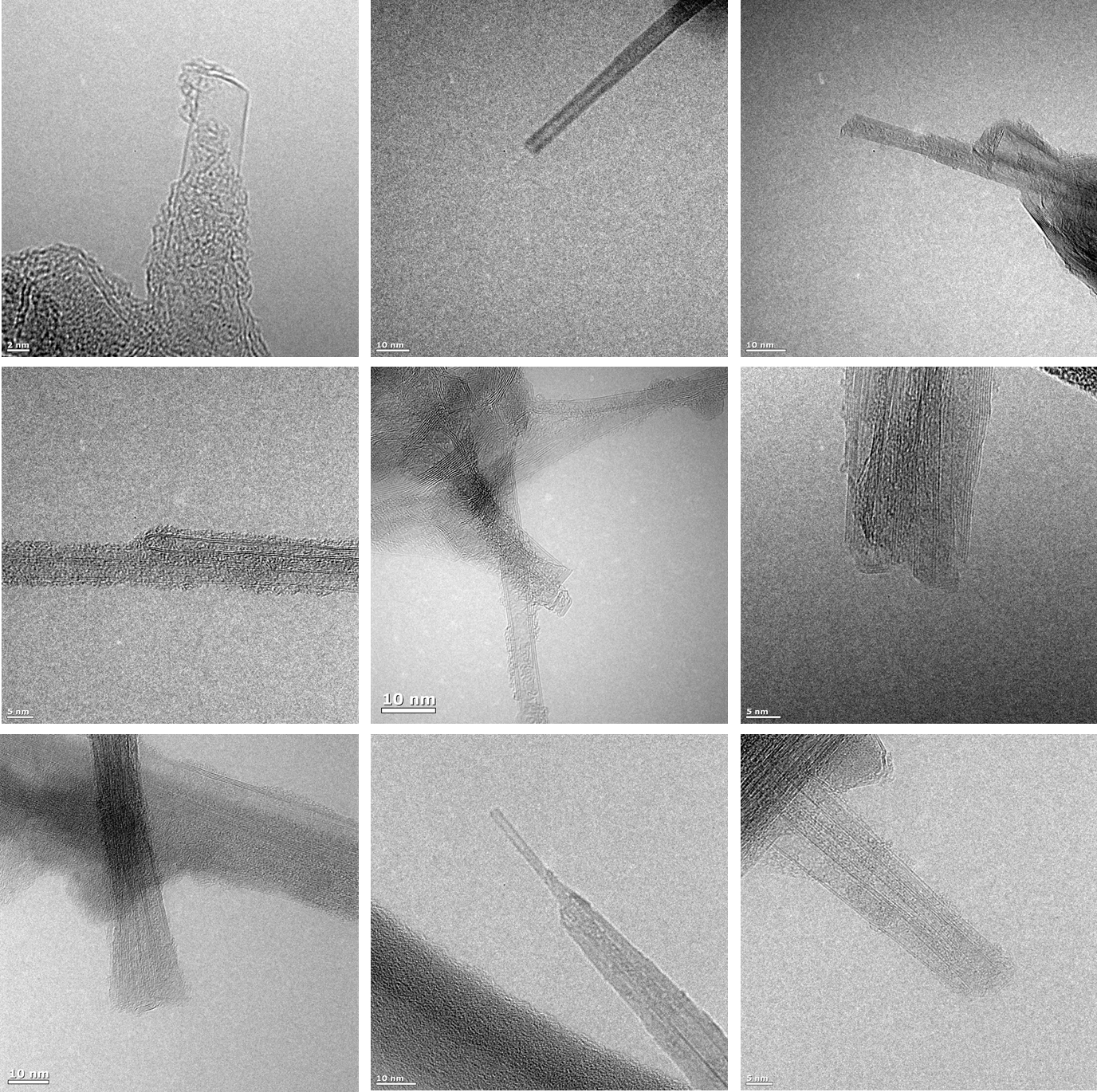


Figure S3. Structure of tube ends analyzed by HR-TEM. Most of the tube ends are closed without boron droplets.


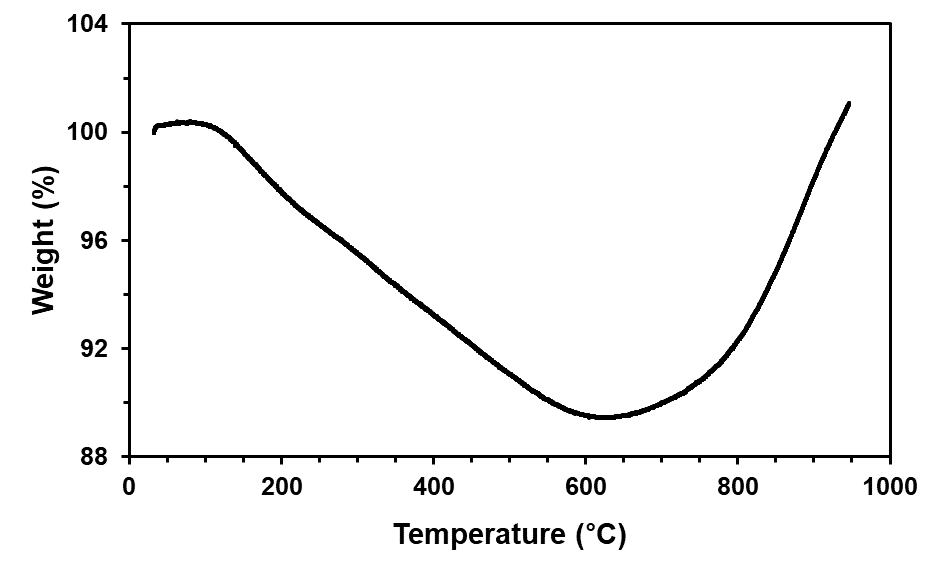


Figure S4. Thermogravimetric analysis of as-grown BNNTs. Amorphous boron content is 1.35 wt.%


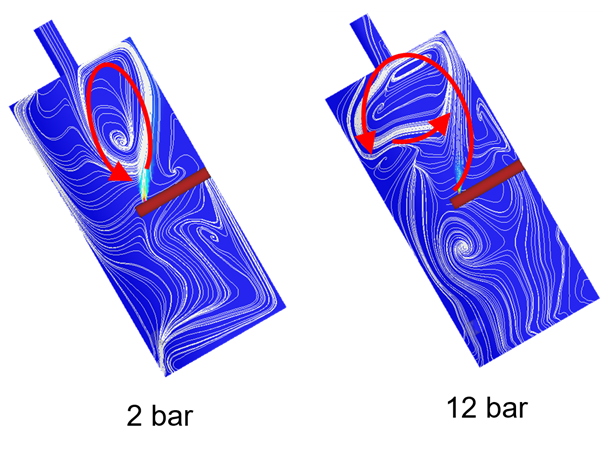


Figure S5. Streamlines calculated in the reaction chamber at (left) 2 and (right) 12 bar. Red arrows indicate a potential pathway of BN precursors or debris formed.
